# A Fast and Low-Cost Human Body 3D Scanner Using 100 Cameras

**DOI:** 10.3390/jimaging6040021

**Published:** 2020-04-09

**Authors:** Mojtaba Zeraatkar, Khalil Khalili

**Affiliations:** 1 Department of Mechanical Engineering, University of Birjand, Birjand 9717434765, Iran; kkhalili@birjand.ac.ir; 2 Department of Mechanics, Mathematics and Management, Polytechnic University of Bari, 70126 Bari, Italy; 3 Institute for Biomechanics, ETH-Zurich, 8093 Zurich, Switzerland

**Keywords:** 3D scanning, human body scanning, image-based scanning, low-cost scanners, structure from motion, raspberry pi

## Abstract

The human body is one of the most complicated objects to model because of its complex features, non-rigidity, and the time required to take body measurements. Basic technologies available in this field range from small and low-cost scanners that must be moved around the body to large and high-cost scanners that can capture all sides of the body simultaneously. This paper presents an image-based scanning system which employs the structure-from-motion method. The design and development process of the scanner includes its physical structure, electronic components, and the algorithms used for extracting 3D data. In addition to the accuracy, which is one of the main parameters to consider when choosing a 3D scanner, the time and cost of the system are among the most important parameters for evaluating a scanner system in the field of human scanning. Because of the non-static nature of the human body, the scanning time is particularly important. On the other hand, a high-cost system may lead to limited use of such systems. The design developed in this paper, which utilizes 100 cameras, facilitates the acquisition of geometric data in a fraction of a second (0.001 s) and provides the capabilities of large, freestanding scanners at a price akin to that of smaller, mobile ones.

## 1. Introduction

3D scanning technology has numerous applications, from micro-scale features [1,2] to large-scale objects such as buildings [3,4]. Human body scanning is one of the most complicated applications in terms of modeling due to the more complex features of the human body compared to other objects as well as to displacements which occur during the scanning [5,6]. Human body scanning has various applications in fields such as medicine, sports, the garment industry, movies and animation, security, and sculpture. In the past decades, technological advances have enabled diagnostic studies to reveal more detailed information about the internal structures of the human body. X-rays, magnetic resonance imaging (MRI), computed tomography (CT), and ultrasounds are commonly used to study physiology and anatomy in vivo and to aid in the diagnosis and monitoring of a multitude of disease states [7]. However, in some applications, external measurements of the body are also important. Medical professionals widely use size, shape, texture, color and skin surface area to assess nutritional status and developmental normality, to diagnose numerous cutaneous disease, and to calculate the requirements for drug, radiotherapy, and chemotherapy dosages; body measurements are also used for the production of prostheses [8,9,10]. From a medical perspective, 3D scanning applications can be divided into four groups: epidemiology, diagnosis, treatment, and monitoring [11]. Detection of the shape of the back in scoliosis patients [12,13], evaluation of human skeleton changes in daily life [14], and evaluation of the effect of sports on the human body [11] are examples of 3D scanning in healthcare applications. The extraction of the features of body organs and their reproduction is an example of a 3D scanning application in biomedical engineering [15]. Identifying human facial expressions [16,17] and predicting face attractiveness [18] are other examples of this technology’s applications in scanning parts which are smaller than the human body as a whole. The use of 3D scanning in clothing design is another interesting application that is used widely in tailoring [19,20,21].

Generally, 3D scanning technologies for body scanning include laser technology, structured light, millimeter waves, infrared waves, and stereophotogrammetry [5]. Each technology has its own costs, advantages, and limitations. Unlike in the industrial sector, accuracy is not the main parameter to consider when choosing a scanner in the field of human scanning. In most of these applications, accuracy has been identified from tenths of millimeters to below 10 mm [10,22], except in some cases such as movies and animation, when higher accuracy is required [23]. Indeed, in body scanning, large errors occur due to the non-static nature of the human body and to the occurrence of displacements during the scanning process, such as small movements, breathing, and blinking. For example, just during quiet breathing in healthy subjects, the breathing amplitude is one-third of a deep breath (approximately 3 cm) based on the average 3D distances of the chest and abdominal wall [24]. These displacements might be different from one person to another, depend on age, posture, and sex [25], lead to a change in the position of the human body, and cause errors in the final 3D model. To avoid large errors, the scanning time has to be as short as possible, in order to freeze the scene. On the other hand, if a technology has a very high cost, its applications in various fields related to human body scanning, like the garment industry, will likely be limited [23]. Thus, achieving a very fast scanning procedure and a low cost of 3D scanners is the main goal of our work.

Table 1 provides an overview of the costs and capabilities of several scanners which are used to scan the human body. They range from lower-cost scanners, which should move around the object or require the object to move (high required time for scanning), to expensive scanners that can capture the body from all sides simultaneously. In 1998, for the first time, the companies Cyberware and Vitronic applied laser-based scanners to extract a 3D model of the human body [26]. In these systems, laser beam variations are viewed using cameras under a fixed angle. Cyberware WBX and Vitronic smart XXL are the newest models released by these companies, providing higher resolutions at a reduced price compared to previous ones. In laser-based systems, a laser line is usually used to sweep the objects surface [27]; however, to speed up the process, multilines or structured light with different patterns have been used to extract the geometrical parameters of the object in structured light systems. The pattern could be in the form of dots, bars, or other elements. Because the recorded light pattern is more difficult to convert into 3D dimensions in comparison with that of a laser-based system, the accuracy and resolution of these systems are lower than those of laser-based ones [5]. Artec, SpaceVision, and SizeStream, among others, are the most famous companies that use structured light to scan the human body (Table 1). The introduction of the Kinect scanner by Microsoft Co., used along with X-box devices, has led to a renewed interest in 3D scanners. Kinect is a structured light-based scanner; however, the hardware and technology used in the Kinect depend on the version of the system. At first, Kinect systems were used for game computers, wherein they tracked the movement of a human body. After development, several companies, such as TC2 and Sizestream, started using this system in their body scanners [23]. Unfortunately, Microsoft Co. stopped supporting the Kinect scanner, therefore updates or future support will be unavailable [28]. In these systems, the maximum resolution when using four Kinect systems was 5 mm [29]. Various research groups have used a sense scanner (manufactured by 3D Systems) for body scanning, which is an inexpensive hand-held scanner. As in the case of Kinect, the sense system has an IR projector, an IR detector, and a camera that senses the colors of the object. Mendricky and Maly [28] designed and developed a rotary body scanner using a sense scanner. This system, which is a low-cost scanner (approximately $1500), provides a maximum deviation range of up to 1 mm for static subjects. However, for a non-static object, the accuracy of the final model is far beyond the accuracy that can be obtained with one-shot whole-body scanners.

Recovering by using two or more cameras is another method which can be used to create a 3D model of the human body [30]. Stereophotogrammetry is the most popular method among the image-based extraction methods for which camera calibration is necessary. The calibration is a time-consuming process, especially when it is necessary to scan a body from different points of view [31]. Stereo may be a suitable method for rigid and static objects but it is not practical in the case of human body scanning. Extracting a geometrical model of the human body using multiple single cameras is called “structure from motion” [32,33]. In this method, extraction of the 3D geometry is carried out using a set of images which had been taken without camera calibration, with no information about the position, direction, and internal parameters of the cameras, such as focal length [34,35]. In recent years, some photogrammetry-based scanners for body scanning have been developed, like a novel body scanning system using 119 cameras [10], which is an expensive standing scanner for healthcare applications [36]. This system is a fast scanner that yields at its best functionality an average error of 0.21 ± 1.27 mm in the case of a static mannequin. Straub and Kerlin [22] presented a photogrammetric full body scanner using 50 inexpensive cameras, which is able to scan human subjects with an average error of 9.4 mm (0.37 in). The designed scanner in this paper uses the structure-from-motion method to extract a 3D model of the human body. This system is a relatively low-cost, very fast, and high-accuracy 3D full body scanner. In addition to the advantages of a passive system, such as not requiring a preplanned scene with a dedicated lighting system and design, there is no need for the time-consuming process of camera calibration. On the other hand, because it uses typical inexpensive cameras and lighting sources, the cost of the present system is much lower than that of laser-based and structured light-based systems. The present scanner is an improvement on the state of the art since, to the best of the author’s knowledge, a 3D body scanner with such a high number of cameras is neither described in the research literature nor being used commercially. Consequently, the design and architecture of the scanner are innovations of the present paper, alongside the embedded high accuracy resulting from the high number of cameras and the induced hyper-redundancy of the scanning system [37]. The information required for a scan of the body can be obtained in less than a second, with comparable functionality to scanners costing between $200,000 and $240,000.

This paper provides information on the materials and methods in the design and development of the 3D scanning system. The design process for creating a 3D scanning system can be categorized into three general sections, including the design and creation of the physical structure, of the electronic components, and of the software. Then, an overview of the system operations and the results are discussed, before the paper is concluded.

## 2. Materials and Methods

**Figure 1:**
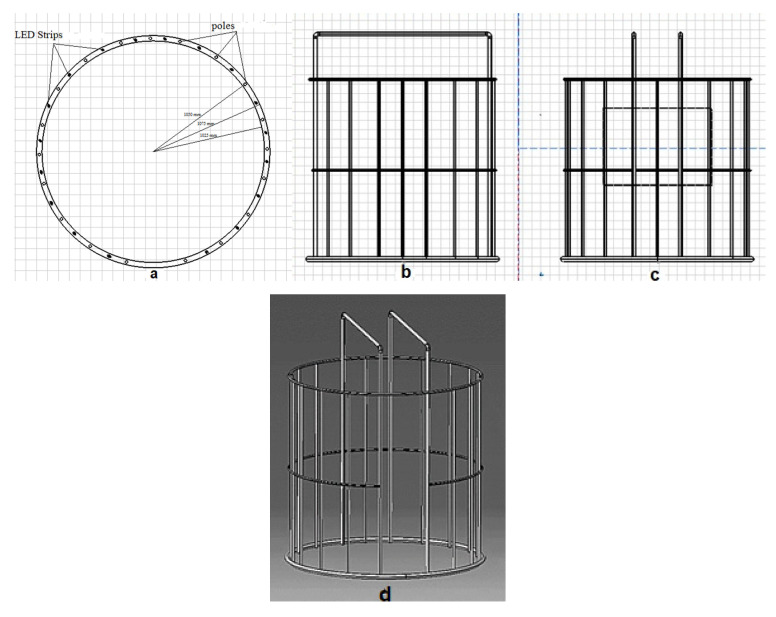
Physical structure of the scanner. (**a**) Top view: steel rods located at 105 cm from the center of the bottom circle. (**b**) Right view: 19 steel rods used for holding the controllers and cameras at specified distances. (**c**) Front view: the angle of the poles on the bottom ring are different from each other. This angle is 15° for the front and sides views of the subject and 20° for the other poles. (**d**) Perspective view of the physical structure.

The designed 3D scanner is a 2D image-based scanner, which is used to extract 3D information of the human body using the structure-from-motion technique. To do this, at first, several images should be captured from different angles around the given object. Raspberry pi cameras and relevant controllers (Raspberry Pi 3 Model B+, Raspberry Pi Foundation, Cambridge, UK) were utilized to capture the images and transmit them at high speed to the main server. The controllers, together with cameras, were placed in specified positions around the object. Figure 1 presents the physical structure of the scanner, fabricated with stainless steel. As shown in Figure 1, the physical structure includes three rings with an inner diameter of 105 cm, called the top, middle, and bottom rings and formed using a rolling process. A total of 100 cameras and relevant controllers were utilized within the scanner. For holding these modules, 15 stainless-steel poles with a diameter of 25 mm and a length of 200 cm, and 4 stainless steel poles with a diameter of 25 mm and a length of 250 cm were used (Figure 1b–d).

The poles are placed about 105 cm from the center of the bottom ring (Figure 1a), providing enough space for a person to enter and stand within the scanner. The other ends of the poles are fixed at the top ring (Figure 1c,d). The middle ring is to provide more rigidity to the structure and a reduced level of movement between cameras and the object during image capturing (Figure 1d).

**Figure 2:**
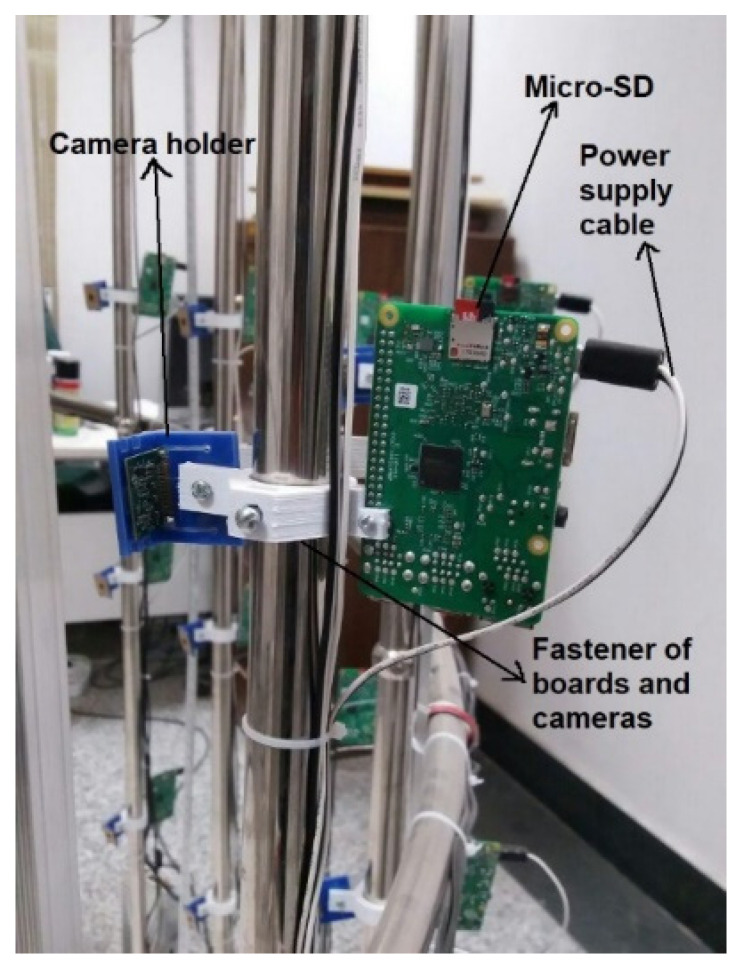
Fastener and holder of controllers and cameras.

Five cameras and relevant controllers were installed on each of the fourteen 200 cm-long stainless-steel poles (with a distance of 40 cm between each pair of cameras), and six cameras were installed at a specified distance on four stainless-steel poles with a total length of 250 cm, with a stainless-steel pole having a length of 200 cm placed in front of the object. In total, 100 cameras were employed for the whole scanner. To fix cameras and controllers on the stainless-steel poles, vibration-absorbent fasteners and holders were used, as shown in Figure 2. These parts were made of PLA (Polylactic acid), using 3D printing technology.

The electronic components of the 3D scanning system incorporate the following items:

- The main server (Quad-core Intel Core i7, 16 GB RAM, 2 GB GPU, ASUS, Taipei, Taiwan)
- 100 Raspberry pi controllers (Raspberry Pi Foundation, Cambridge, UK)
- 100 Raspberry pi cameras (Raspberry Pi Foundation, Cambridge, UK)
- 100 8 GB external storage cards (SanDisk, Milpitas, CA, USA)
- A wireless router (Linksys EA6300, Linksys, Irvine, CA, USA)
- A lighting system
- Adjustable power supplies

The main server is a computer which provides command transmission to/from the controllers using a Linksys router. This server is also used for processing of the images. The cameras are the main components of the electronic part of the scanner. Cameras with a resolution of 5 megapixels were installed on the physical structure in specified positions by considering that an accurate 3D reconstruction requires at least 60% overlap between two adjacent 2D images [38]. The cameras were directly connected to the controllers using ribbon cables and could be controlled through the main server using Raspberry pi controllers. Micro-SDs were used to install the corresponding operating system (Raspbian, Jessie 2016, Raspberry Pi Foundation, Cambridge, UK) on the controllers and were also used as a temporary memory for storing the captured images before transmission to the main server.

**Figure 3:**
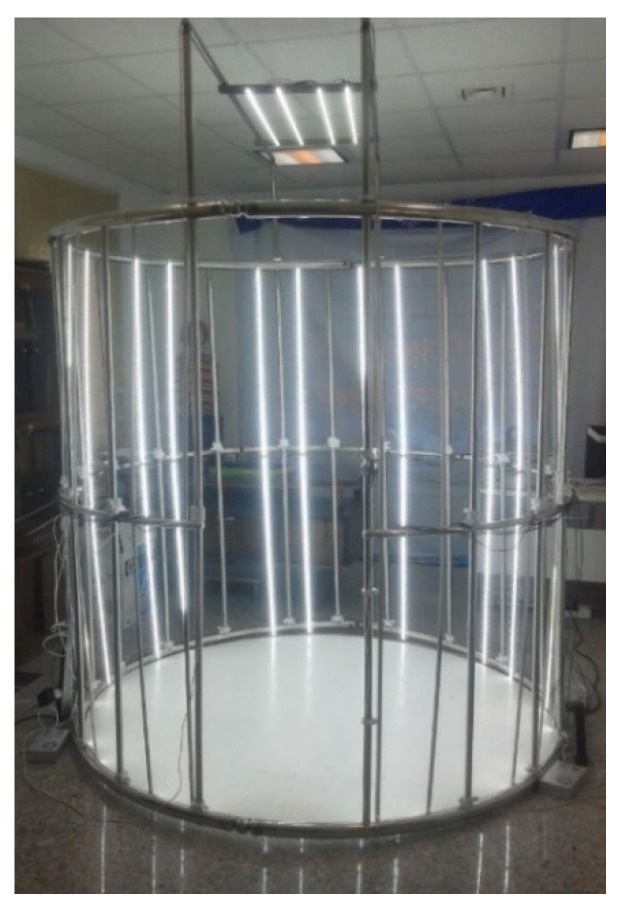
Lighting in the 3D scanning system.

**Figure 4:**
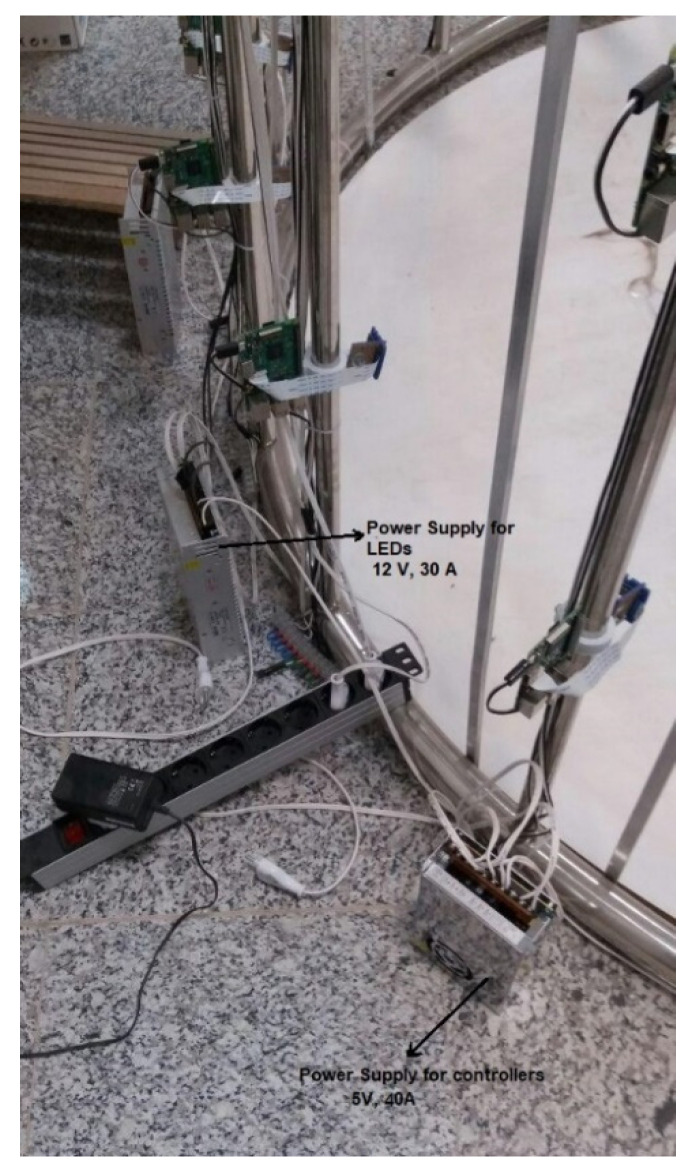
Power supply in the 3D scanning system.

The lighting system is another significant part of the designed scanner. While an image-based system’s lighting is not as important as it is for active systems, it is still vital to provide evenly distributed light throughout the scene. The lighting system plays an important role in determining the quality of the images and the precision of the final model [39]. In this study, various lighting systems were investigated and, finally, as shown in Figure 3, strip LED (Light-Emitting Diode) lights were used that illuminate the scene from the sides and from the top. The lighting system was designed to provide lighting at three different illuminances levels: low, medium, and high. Several power supplies were used to supply electricity for the Raspberry pi controllers (see Figure 4). An input voltage of 5 V and a current of 2.5 A are needed for each controller. The input voltage and current were 12 V and 2 A per each meter of LED lights.

The architecture of the software is such that it includes two main parts: the control part (the driver of the system) and the processing part that provides some image processing, image registration, stitching, and so forth. The camera controlling portion includes operations such as sending commands by the operator, receiving and performing commands by the controllers, sending captured images to the main server, and saving images in a specified file. All these operations are undertaken by the software automatically. The captured images are processed by various algorithms in several steps to extract the 3D point cloud of the object. In the structure-from-motion method there is no need for calibration, and internal and external parameters of the camera are extracted via captured images during self-calibration [40]. 

**Figure 5:**
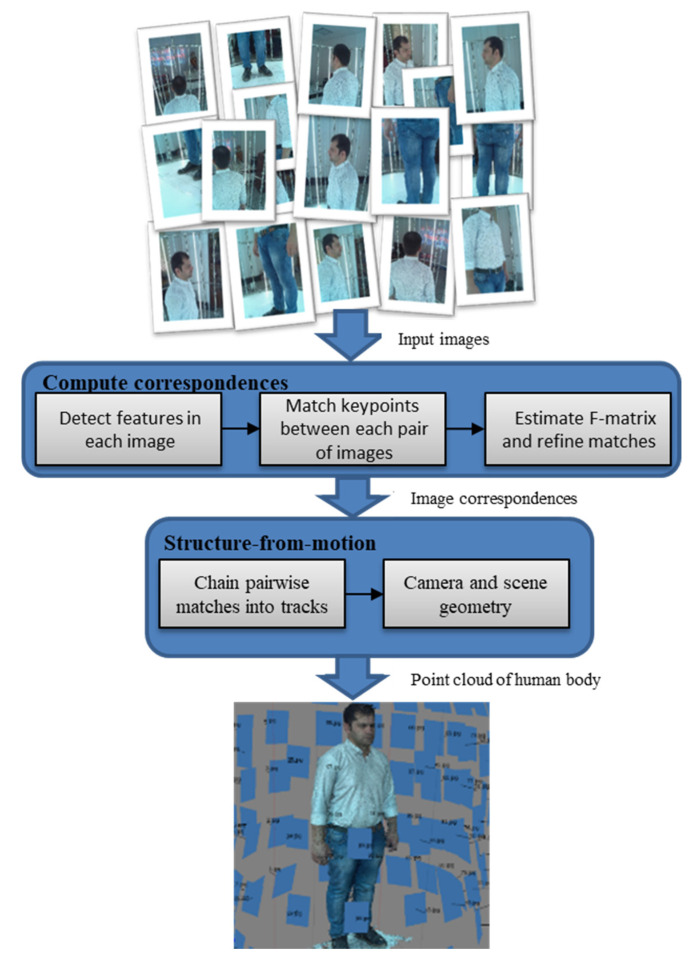
Block diagram of image processing in the 3D scanning system.

The pipeline of image processing is shown as a block diagram in Figure 5. The first step of image processing is detecting key points of each image and extracting appropriate features to match with key points of other images. In the present study, because of its good invariance to image A typical transformations, the SIFT (Scale-invariant feature transform) key point detector algorithm [41] was applied. The image contained several thousand SIFT key points. The SIFT algorithm provides a local descriptor for each key point. Following key points detection, image registration was performed between each pair of images, and this was done based on the approximate nearest neighbors of feature vectors. A KD-tree (K-dimensional tree) was used to find the closest neighbor of a set of feature points [42].

In the next step, after matching the features for an image pair, the RANSAC algorithm [43] was used to estimate the fundamental matrix (F matrix) and remove spurious matches. To estimate the F matrix and determine true matches out of spurious matches, the RANSAC algorithm uses eight random points in each pair of input images [44].

When consistent matches were detected in each pair of images, the VisualSFM (V0.5.26, 2013 ) package [45] was used to extract camera parameters and the 3D point cloud using the structure-from-motion technique. In this method, at first, detected correspondent key points among various images are divided into some tracks (a track is a connected set of matching key points across multiple images) so that each set corresponds to a point in the 3D scene. In other words, the image of a point in the scene is tracked in various images, and this set of key points is considered as a track. The recovery of the 3D structure of a scene using a set of tracks is the subject of a structure-from-motion problem. The basis of the structure-from-motion method is an optimization problem. In fact, we look for a setting of cameras and 3D points of the scene which are in the best agreement with the detected correspondents according to the projection equations [45,46].

## 3. Results and Discussion

**Figure 6:**
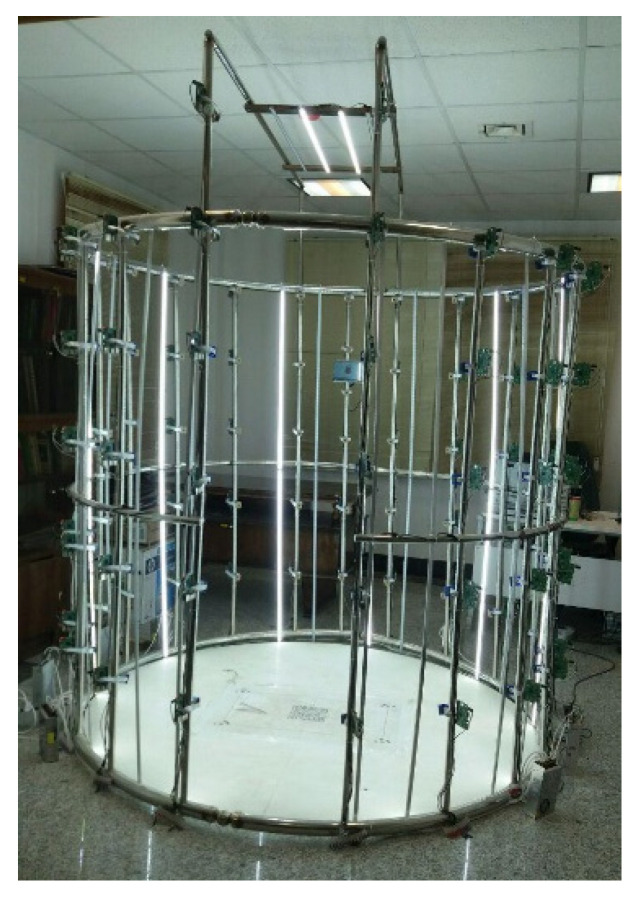
Developed 3D scanning system.

This paper presents the design and development of a fast and low-cost 3D scanner for human body scanning. Figure 6 shows an overview of the developed 3D scanner. The physical structure of the scanner was designed according to the human body; however, it can be used for scanning both inanimate objects and humans. The measuring range of the scanner is 35 × 35 × 200 cm in X-, Y-, and Z-axes, respectively, with regards to the designed physical structure and using raspberry pi cameras. The hardware costs of the scanner, including the camera units, frame construction, main server, lighting system, and networking, are shown in Table 2. The designed 3D scanner uses 100 inexpensive typical cameras to extract the geometric parameters of a human body. A major part of the cost of the system is related to the cameras; therefore, it could be said that the cost of the developed scanner (approximately $6000) is much lower than that of laser-based and structured light scanners, which can capture all sides simultaneously (see Table 1).

**Figure 7:**
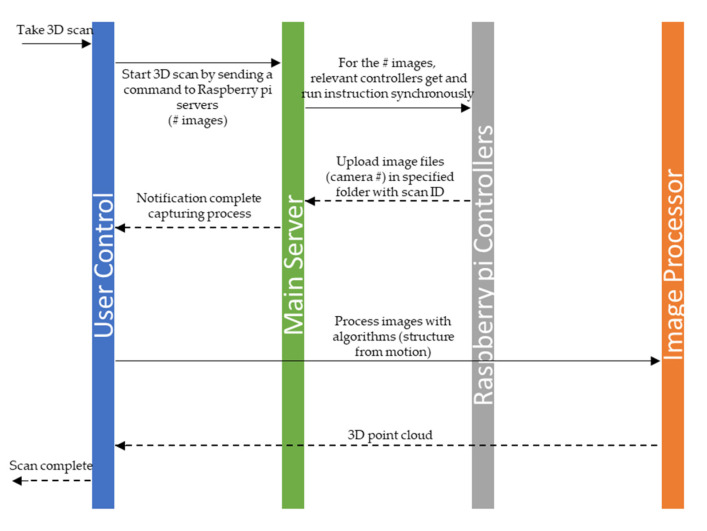
Sequence diagram of operation in the developed 3D scanner.

**Figure 8:**
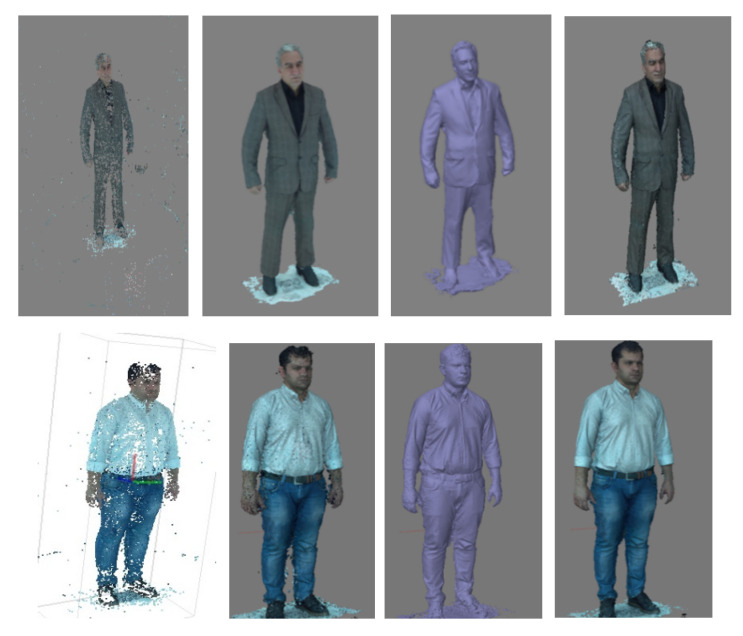
Examples of 3D modeling of the human body. From left to right: Tie-point cloud; dense-point cloud; triangle mesh; 3D model with texture.

The scanning process begins with collecting images from different angles around the given object. In this design, camera locations and distances were chosen to ensure significant overlap (60%) between the images by providing 40 cm between each camera and different angles of poles relative to each other in the length and width directions, respectively. Figure 7 shows the unified modeling language (UML) sequence diagram of operation in the 3D scanner. The process of capturing images is done by sending a user command to the raspberry pi units through the main server. This message is received by each raspberry pi unit (a delay is introduced by the wireless router which can be eliminated by synchronous execution of the command by the raspberry pi units), which then collects images within a fraction of a second and uploads them to the main server. Owing to the non-rigidity of the human body and to displacements during the scanning process, the capturing time is an important parameter in the scanning of a human body. Small and mobile 3D scanners (e.g., Sense, Artec EVA) need a considerable amount of time to extract geometrical information by rotating around the subject or having the subject move [6]. Table 3 compares the required time for data acquisition by the designed scanner and by some freestanding and mobile scanners. The time required for the well-known laser-based and structured light scanners Cyberware WBX, Vitronic smart XXL, and SpaceVision is 17, 12, and 2 s, respectively. Long scanning times lead to a change in the position of the body, causing errors in the reconstructed 3D model. Image-based full-body scanners provide faster speeds; however, these systems require a large number of sensors to capture all sides simultaneously, which in turn increases the final cost of the scanner. The system developed here uses 100 cameras that facilitate the acquisition of the geometric and texture data in 1 millisecond and offers the capabilities of large, freestanding photogrammetric scanners (3dMDbody-Flex8, INBODY) at the price of small, handheld scanners. The acquisition time is fast enough to freeze the scene and eliminate errors in the reconstructed model, which would otherwise exist due to displacements during the scanning process. In comparison to our rotary-type scanner [6], the developed 3D scanner with a higher speed provides a higher quality of results in terms of the detail and accuracy of the final model (Figure 8).

In addition to the elimination of displacements in the developed scanner, the very fast scanning procedure allows it to be used as a newly developed breathing movement-measuring device. Physical assessment of breathing is an important indicator of a person’s health. Respiratory rate is a vital sign used to monitor the progression of illness, and an abnormal respiratory rate is an important marker of serious illness [47]. Several studies have shown that the respiratory rate is better than other vital measurements, such as pulse and blood pressure, in discriminating between stable patients and patients at risk [48]. Little knowledge is available on the visual aspects of breathing, including the respiratory muscle movements, breathing pattern, and breathing volume. There are several contact-based and contactless methods to accurately measure chest and abdominal wall movements; however, because of their high cost and the requirement for well-trained technicians, these methods are rarely used in clinical practice [24,49]. Thus, to assess chest wall mobility, chest excursion has typically been measured using a tape measure [50], which is a simple and inexpensive method but cannot be used to evaluate any asymmetry or abnormality in chest and abdominal wall mobility. The 3D scanner developed in the current work can be used as a useful research tool to measure breathing movements in healthcare applications.

**Figure 9:**
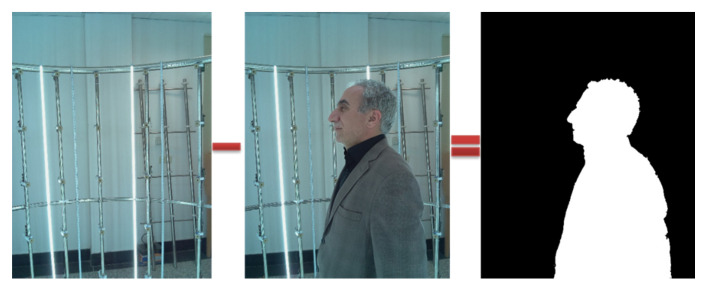
Removing the background by capturing two images from a single viewpoint (with and without the object).

In image-based 3D reconstruction, the background of the image has a direct impact on the calculation time, precision, and quality of the final model. In this paper, the backgrounds of the images were removed using two sets of images (with and without the human figure). Therefore, in general, we captured 200 images and sent them to the main server. The backgrounds of the images were removed by subtracting these two sets of images (Figure 9).

To extract the 3D point cloud, the captured images were processed in several steps using the various algorithms introduced in Section 2. Figure 8 shows the point cloud of a human body. The required time for processing images is a function of the main server specifications, number of input images, and level of available texture in images. This time is approximately 30 min for 100 images with a medium level of texture and main server specifications (Quad-core Intel Core i7, 16 GB RAM, 2 GB GPU). Geomagic Studio software was used to generate meshes and surfaces from the 3D point cloud. It is possible to export an output with an arbitrary format from the software to be read by other commercial reverse engineering software packages. For instance, it is possible to export an output with *stl or *obj formats that can be used in 3D printers. Texture and color could also be assigned to the 3D model using captured images. The textured 3D models could be useful monitoring tools for numerous cutaneous illness.

The application of scanners which can capture all sides simultaneously is limited by their high cost. Although a high cost is acceptable in some fields, such as the medical and movie and animation fields, it is not acceptable in other fields. It seems that a reduction in the final cost of a scanner could lead to a wider application of these devices in various fields related to human body scanning. It must be noted, however, that because of the use of special lighting (for example laser beams), some scanners may be hazardous or dangerous for humans. Individuals typically prefer not to be exposed to laser radiation. Hence, image-based scanners are more acceptable for human scanning.

## 4. Conclusions and Future Work

This research presented the design and development of a very fast and low-cost human 3D scanner. The paper described the physical structure, electronic components, and different algorithms used for the design and development of the scanner and provided an overview of the process of its construction. The described scanner has applications in various fields related to human body scanning, particularly in areas where existing technologies are less commonly used because of their high cost (e.g., the garment industry). This scanner could be a useful research tool across a wide variety of prospective projects, including data collection. With a cost of approximately $6000, it is designed to enable significant additional research applications. Future work will include the characterization of the accuracy and performance of the scanner under various configurations.

## Figures and Tables

**Table 1:** Costs and capabilities of scanners used for human scanning.

Scanner	Type	Cost	Time of Scanning
3D Systems Sense ^1^	Optical subject or scanner must move	$419	Depends on subject/operator
Artec EVA ^2^	Optical subject or scanner must move	$19,800	Depends on subject/operator
Geomagic Capture ^3^	Optical with LED point emitter Subject or scanner must move	$14,900	Depends on subject/operator
Gotcha 3D Scanner ^4^	Optical subject or scanner must move	$10,000	Depends on subject/operator
Head & Face Color 3D Scanner (Model 3030/RGB/PS)—(CyEdit+) ^5^	Laser Fixed scanner/fixed subject	$63,200	Not specified
Head & Face Color 3D Scanner (Model 3030/sRGB/PS)-Hires Color—(CyEdit+) ^6^	Laser Fixed scanner/fixed subject	$73,200	Not specified
Head & Face Color 3D Scanner (Model PX)—Single View—(PlyEdit) ^7^	Laser Fixed scanner/fixed subject	$67,000	Not specified
Head & Face Color 3D Scanner (Model PX/2)—Dual View—(PlyEdit) ^8^	Laser Fixed scanner/fixed subject	$77,000	Not specified
Vitronic-Vitus Smart XXL ^9^	Laser-fixed subject	$65,000	12 s
KX-16 3D Body Scanner ^10^	Infrared subject or scanner must move	$10,000	7 s
IIIDBody ^11^	Optical fixed subject and scanner	$20,000–50,000	Not specified
SizeStream-3D Body Scanner ^12^	Infrared	$15,000–20,000	6 s
SpaceVision-Cartesia ^13^	Laser structured light-fixed subject and scanner	$20,000	2 s
INBODY	Photogrammetric full-body scanner	Not specified	0.05 s
3dMDbody-Flex8 ^14^	Stereophotogrammetry	$190,000	0.002 s
Whole-Body 3D Scanner (Model WBX)—(DigiSize Pro) ^15^	Laser line-fixed subject	$200,000	17 s
Whole-Body Color 3D Scanner (Model WBX/RGB)—(DigiSize Pro) ^16^	Laser line-fixed subject	$240,000	17 s

^1 ^https://www.3dsystems.com/shop/sense; ^2 ^https://www.artec3d.com/handheld-3d-scanners/artec-eva; ^3 ^https://gomeasure3d.com/geomagic-capture/; ^4 ^https://thinglab.com.au/3d-scanners/3d-scanners-stationary/mephisto-gotcha/; ^5 ^https://www.yumpu.com/en/document/view/37026451/head-face-color-3d-scanner-cyberware; ^6 ^https://www.yumpu.com/en/document/view/37026447/model-3030-color-3d-scanhead-cyberware; ^7 ^https://www.yumpu.com/en/document/view/37026456/head-face-3d-scanner-model-px-cyberware; ^8 ^https://www.yumpu.com/en/document/view/37026456/head-face-3d-scanner-model-px-cyberware; ^9 ^https://www.vitronic.de; ^10 ^https://www.tc2.com/; ^11 ^https://www.aniwaa.com/product/3d-scanners/4ddynamics-iiidbody/; ^12 ^http://www.sizestream.com/; ^13 ^http://www.spacevision.tokyo/; ^14 ^http://www.3dmd.com/; ^15 ^https://www.sciencedirect.com/topics/engineering/cyberware; ^16 ^https://www.sciencedirect.com/topics/engineering/cyberware.

**Table 2:** Hardware costs of the developed human body 3D scanner.

Component	Quantity	Unit Price	Total *	Possible Suppliers
Raspberry Pi 3 Model B with external storage	100	$34.5	$3450	https://www.newark.com/buy-raspberry-pi?ost=raspberri+pi+3+model+b&rd=raspberri+pi+3+model+b
Pi Camera	100	$14	$1400	https://www.amazon.com/Camera-Module-Raspberry-Atomic-Market/dp/B075DKDGPF
Frame construction	1	$300	$300	
Main server	1	$800	$800	
LED strips full spectrum 18 × 2 m + 4 × 1 m	18 + 4	$9.5	$380	https://www.alibaba.com/product-detail/Full-spectrum-LED-grow-strip-warm_60666295850.html?spm=a2700.7724857.normalList.118.1c39449dZvYvbn
Power-supply 5V, 30A	4	$23	$92	https://www.amazon.com/LETOUR-Converter-200Watts-Adapter-Lighting/dp/B07FX8HL79
Led Power-supply 12V, 30A	4	$18.95	$75.8	https://www.amazon.com/eTopxizu-Universal-Regulated-Switching-Computer/dp/B00D7CWSCG
Ethernet Switches	1	$50	$50	https://www.linksys.com/ae/p/P-EA6300/#product-features

* Prices exclude build cost and VAT.

**Table 3:** Time required for data acquisition by different human body 3D scanners.

Scanner	Time Required (s)
Sense	High: depends on subject/operator, scanner or subject should be rotated
Artec EVA	High: depends on subject/operator, scanner or subject should be rotated
Cyberware Whole-Body Color 3D Scanner	17
SizeStream-3D Body Scanner	6
Vitronic-Vitus Smart XXL	12
SpaceVision-Cartesia	2
INBODY	0.05
3dMDbody-Flex8	0.002
Designed scanner	0.001
